# Prevalence and Awareness of Anterior Cruciate Ligament Injuries Among Full-Contact, Semi-contact, and Non-contact Sports Athletes in the Kingdom of Bahrain

**DOI:** 10.7759/cureus.65180

**Published:** 2024-07-23

**Authors:** Joud K Alsaeed, Salman S Salman, Khalid J Alsuwat, Abdulrahman A Aldoseri, Salah A Mustafa, Rayan A Alzahrani, Ahmed M Alasmari, Jasim K Aljasim, Ayman Y Alsaffar, Abdulla A Aljowder, Yahya M Naguib

**Affiliations:** 1 Training and Academic Department, Salmaniya Medical Complex, Manama, BHR; 2 Orthopedics Department, Al Hada Armed Forces Hospital, Taif, SAU; 3 Pediatric Department, Bahrain Defence Force Hospital, Riffa, BHR; 4 Orthopedics Department, Bahrain Defence Force Hospital, Riffa, BHR; 5 General Directorate of Health Affairs, Ministry of Health of Saudi Arabia, Jeddah, SAU; 6 General Directorate of Health Affairs, Ministry of Health of Saudi Arabia, Aseer, SAU; 7 Orthopedics Department, King Hamad University Hospital, Muharraq, BHR; 8 Physiology Department, College of Medicine and Health Sciences, Arabian Gulf University, Manama, BHR; 9 Clinical Physiology Department, Faculty of Medicine, Menoufia University, Shibin El Kom, EGY

**Keywords:** preventive training, awareness, injury, exercise, anterior cruciate ligament

## Abstract

Background

The anterior cruciate ligament (ACL) is a crucial connective tissue that links the femur to the tibia, playing a vital role in stabilizing the knee by resisting forward and rotational movements. ACL tears can occur due to both contact and non-contact sports injuries. Diagnosis and assessment typically involve the Lachman test and magnetic resonance imaging. Initial treatment focuses on reducing swelling, followed by physical therapy or surgery to restore long-term knee functionality.

Objective

This study aimed to assess the injury prevalence and awareness of ACL and the engagement of preventing training programs among Bahraini athletes across diverse sports.

Methods

A cross-sectional survey was utilized to evaluate injury prevalence and awareness of ACL and engagement in preventing training among 161 Bahraini athletes from different sports. Data were collected through a detailed questionnaire addressing demographics, sports involvement, ACL injury history, and preventive training. The analysis involved descriptive statistics, one-way ANOVA, and independent t-tests to compare knowledge across sports types. A chi-square test was performed to examine correlations between injury history and training.

Results

The results showed that athletes in full-contact sports exhibited significantly higher ACL injury awareness compared to those in semi-contact and non-contact sports. Furthermore, a higher percentage of athletes in semi-contact and non-contact sports reported a history of ACL injuries when compared to those in full-contact sports. However, there was no significant difference in the engagement of preventive exercise training across different sports categories.

Conclusion

A notable gap in ACL injury awareness exists among Bahraini athletes, varying significantly across sport types. The data highlight the need for customized educational programs catering to different sports.

## Introduction

The anterior cruciate ligament (ACL) is a strong fibrous tissue within the knee joint that provides significant resistance to sliding and twisting motions. It is an essential component for knee stability, which relies on the harmony between static and dynamic structures [1,2]. Athletes who suffer ACL tears often experience concurrent damage to the meniscus and cartilage, which are part of a classification including direct, indirect, and non-contact types of ACL tears. Direct ACL tears result from knee impact, commonly observed in collisions during sports like football. By contrast, indirect tears occur without direct knee contact, for example, during landing from a jump, while non-contact tears manifest during abrupt changes in direction, as exemplified by a soccer player changing direction while running [3,4].

The annual incidence rate of ACL tears, adjusted for age and sex, stands at one per 3500 persons [5]. In the United States, ACL injuries pose a significant health concern, notably impacting young athletes, with over 100,000 cases reported each year [6]. ACL is commonly injured in elite female athletes up to eight times more likely than their male counterparts. Factors such as anatomical variances, hormonal influences on ligament elasticity, biomechanical dynamics, and potential effects of oral contraceptives contribute to higher ACL injury rates in females [7,8].

The assessment of ACL laxity commonly employs a variety of tests, including the Lachman, Maclintoch, anterior drawer, flexion rotation drawer, Slocum, and Losee tests [9]. Evaluating the integrity of the ACL typically involves the anterior Lachman, anterior drawer, and pivot-shift tests. Each of these tests is distinguished by their individual methodological approaches, evaluation standards, limitations, and accuracy [10]. Notably, the Lachman test emerges as the most precise clinical method for detecting ACL tears, with magnetic resonance imaging serving as the primary diagnostic tool [11]. Following an ACL injury, immediate management aims at reducing swelling through ice application, elevation, and compression. Subsequent treatment typically involves a regimen of physical therapy or surgery to restore knee functionality and mobility [9].

Preventive training programs (PTPs) are recommended to improve balance, lower extremity biomechanics, muscle activation, functional performance, strength, and power, while also decreasing landing impact forces [12]. Implementing ACL injury-prevention training programs can improve neuromuscular control and lower extremity biomechanics, reducing the risk of injury training programs should be regularly supervised by individuals skilled in identifying faulty movement patterns to ensure excellent movement quality and provide feedback on exercise technique [13].

This study aimed to assess the prevalence of ACL injuries among Bahraini athletes across diverse sports categories, along with their awareness and engagement in PTPs.

## Materials and methods

Ethical considerations

The privacy and confidentiality of the articipants were maintained by ensuring informed consent and securely destroying materials after the study. Participants' anonymities were preserved, and their right to refuse participation was respected. Ethical approval for this study was obtained from the Research and Ethics Committee of the College of Medicine and Medical Sciences at Arabian Gulf University (E13-PI-11-22).

Study design and setting

This study was a cross-sectional survey conducted from July to October 2023 in the Kingdom of Bahrain. The study aimed to assess the prevalence and the knowledge regarding ACL injuries. This design was chosen for its effectiveness in evaluating the prevalence and correlations among these parameters within a specified time frame.

Study population and sampling

All participants were recruited from the National Sports Medicine and Rehabilitation Center in the Kingdom of Bahrain, with a total of 161 athletes participating in the study. The study targeted athletes participating in different types of sports including full-contact, semi-contact, or non-contact sports.

Inclusion criteria

Professional athletes who participate in teams officially verified by the Bahrain Athletics Association, with ages between 18 and 35 years, and encompassing both genders, were included.

Exclusion criteria

Athletes who refused to participate, under the age of 18, and had articular cartilage lesions, concomitant lesions of the posterior cruciate ligament, and inflammatory arthritis were excluded.

Data collection tool, methods, and statistical analysis

Data were collected via a structured questionnaire developed to gauge awareness and understanding of ACL injuries [14]. The questionnaire included sections on demographic characteristics, sports category, history of ACL injury, and engagement in PTPs. The questionnaire was distributed in person at the National Sports Medicine and Rehabilitation Center. Descriptive statistics were used to summarize demographic data. One-way analysis of variance (ANOVA) and independent t-tests were applied to compare knowledge levels across different sports categories. A chi-square test was used to assess the relationship between the history of ligament injury and preventive exercise training across all sports categories. A p-value of ≤0.05 was considered statistically significant for all tests. The collected data were analyzed using the IBM SPSS Statistics for Windows, version 26.0 (released 2019, IBM Corp., Armonk, NY). The analysis was performed by members of the research group. Quantitative variables were presented as mean ± standard deviation.

## Results

Table 1 illustrates the general characteristics of the participants in this study. The study included a total of 161 participants, with 24 females (14.9%) and 137 males (85.1%). Full-contact sports including 75 football players, two mixed martial arts (MMA) players, and 18 basketball players had the highest representation, with 95 participants (59.0%), followed by non-contact sports (29.8%), which include 27 paddle players, three volleyball players, and 18 powerlifting players, and semi-contact sports (11.2%), which include 18 handball players. Among the participants, 41 individuals (25.5%) reported a history of ACL injury, while 120 participants (74.5%) reported no previous ligament injury. In terms of anti-ligament injury training, 66 participants (41.0%) reported receiving such training, while 95 participants (59.0%) did not receive any.

**Table 1:** Demographic characteristics of the participants.

Variable	n	%
Gender		
Female	24	14.9
Male	137	85.1
Sport category		
Full contact	95	59.0
Semi-contact	18	11.2
Non-contact	48	29.8
History of anterior cruciate ligament injury		
Yes	41	25.5
No	120	74.5
Preventing training programs (PTPs)		
Yes	66	41.0
No	95	59.0

The sports category was significantly correlated to the history of ligament injury (p-value = 0.029). A higher percentage of participants reported a history of anterior cruciate ligament injury in the semi-contact and non-contact categories compared to the full-contact category (Table 2).

**Table 2:** Relation between sport categories and history of ligament injury. The p-value of the Chi-square of ≤0.05 is considered significant.

History of ligament injury	Sport category			p-value
	Full contact	Semi-contact	Non-contact	
Yes	17 (17.9%)	7 (38.9%)	17 (35.4%)	0.029
No	78 (82.1%)	11 (61.1%)	31 (64.6%)	

Table 3 represents the results of the one-way ANOVA comparing the knowledge about ACL injury among athletes of different sports categories. The data showed that the full-contact category had a significantly higher mean knowledge score (p < 0.0001) when compared to the semi-contact and non-contact sports athletes. The effect size, measured by Cohen's f, was 0.3433, suggesting a moderate practical significance.

**Table 3:** Comparison between the sport categories in terms of their knowledge about anterior cruciate ligament injury. The p-value of the one-way analysis of variance (ANOVA) of ≤0.05 is considered significant. Cohen’s f was used as a measure of the effect size.

Sport category	n (%)	Mean ± SD	p-value	Cohen's f
Full contact	95 (59.0%)	8.97 ± 2.78	<0.0001	0.3433
Semi-contact	18 (11.2%)	8.50 ± 1.95		
Non-contact	48 (29.8%)	6.62 ± 4.07		

Table 4 represents the findings of independent sample t-tests comparing the mean of knowledge scores between pairs of sports categories. When comparing the full-contact and semi-contact categories, data showed insignificant differences in mean scores (p-value = 0.393). A comparison of the full-contact and non-contact categories revealed a significant difference (p-value < 0.0001, Cohen's d = 0.671), indicating that the full-contact category had a significantly higher mean knowledge score with a moderate effect size. Comparing semi-contact and non-contact categories showed insignificant differences between the mean scores (p-value = 0.066).

**Table 4:** Comparison between pairs of sport categories in terms of their knowledge about anterior cruciate ligament injury. * Significant when compared to the non-contact group. The p-value of the independent sample t-test of ≤0.05 is considered significant. Cohen’s d was used as a measure of the effect size.

Variable	n	Mean ± SD	p-value	Cohen's d
Full contact	95 (59.0%)	8.97 ± 2.78	0.393	0.196
Semi-contact	18 (11.2%)	8.50 ± 1.95		
Full contact	95 (59.0%)	8.97 ± 2.78*	<0.0001	0.671
Non-contact	48 (29.8%)	6.62 ± 4.07		
Semi-contact	18 (11.2%)	8.50 ± 1.95	0.066	0.584
Non-contact	48 (29.8%)	6.62 ± 4.07		

There was insignificant difference among study groups in utilizing PTPs (Table 5).

**Table 5:** Relation between sport categories and preventing training programs (PTPs). The p-value of the Chi-square of ≤0.05 is considered significant.

Preventive training programs (PTPs)	Sport category			p-value
	Full contact	Semi-contact	Non-contact	
Yes	33 (34.7%)	10 (55.6%)	23 (47.9%)	0.131
No	62 (65.3%)	8 (44.4%)	25 (52.1%)	

## Discussion

Acute knee injury is often characterized by intense pain and the unmistakable sound of rupture, typically accompanied by joint swelling, limited mobility, muscle weakness, and decreased athletic performance [14]. ACL tears, the most frequently injured knee ligament, account for nearly 50% of all knee injuries [15]. While ACL reconstruction is a common treatment, 85-90% of patients regain normal or near-normal knee joint function within six months post-surgery [16]. However, athletes often report higher levels of knee pain, persistent symptoms, reduced knee function during sports activities, lower quality of life, and diminished objective knee function after ACL [17]. These challenges underscore the importance of ACL injury awareness for effective prevention. Consequently, this study was conducted in the Kingdom of Bahrain to primarily assess the prevalence and awareness of ACL injuries and the effectiveness of PTPs.

Prevalence of ACL

In the present study, the prevalence of a history of ACL injury was 25.5%. This prevalence is higher than the global prevalence of ACL injuries, which ranges between 10% and 25% [18,19]. When compared to the nearby regions, the prevalence of previous ACL injury remained higher than what was observed in the Kingdom of Saudi Arabia, where studies reported rates between 14.7% and 10.3% [18,20]. In India, the prevalence was reported to be 13.5% [21]. The high prevalence in the current study may be influenced by specific factors, such as the type of sport and training practices. According to a study on the basic science of ACL injuries, non-contact sports account for more than 70% of these injuries, primarily due to movements such as landing from a jump or lateral cutting maneuvers [22]. This agrees with the current study, which demonstrated higher prevalence rates of ACL injuries in semi-contact (38.9%) and non-contact sports (35.4%) when compared to full-contact sports (17.9%).

Disparities in ACL injury awareness across sport types

The study revealed significant differences in ACL injury awareness between athletes participating in full-contact, semi-contact, and non-contact sports. Athletes engaged in full-contact sports demonstrated higher levels of awareness, with a mean knowledge score of 8.97, compared to their counterparts in semi-contact (mean score of 8.50) and non-contact sports (mean score of 6.62). This discrepancy suggests that the nature of the sport may influence athletes' understanding of ACL injuries, possibly due to varying injury risk and exposure. Supporting this, a study found that athletes involved in high-risk sports like basketball, football, and boxing had better information about ACL injuries than those in lower-risk sports like cardio [23]. Data indicated a higher proportion of ACL injuries in semi-contact and non-contact sports compared to full-contact sports. This could be attributed to less knowledge about ACL injuries, as athletes in semi-contact and non-contact sports might perceive themselves to be at lower risk and therefore be less informed about preventive measures. Moreover, differences in awareness levels could be influenced by factors, such as gender, age, nationality, and education. A study in Saudi Arabia showed that 56% of athletes had good awareness of ACL tears, with higher awareness significantly associated with males, the 18-25 age group, Saudi nationals, and those with higher education levels [24]. These findings highlight the importance of targeting educational interventions to improve ACL injury awareness across diverse athlete populations.

Effectiveness of PTPs

The study found no significant difference in the prevalence of ACL injuries across different sports categories by athletes reporting participation in PTPs. It was previously reported that comprehensive ACL injury PTPs, including plyometrics, neuromuscular training, and strength training, can reduce injury risk by 52% in female athletes and 85% in male athletes [25]. In addition, a meta-analysis of 13 studies showed a significant reduction in ACL injuries following PTPs [26]. PTPs are generally effective, but our data indicate that inappropriate application of such programs may not be as effective as they should be. One possibility is that traditional training methods focus only on one component exercise. Compared with single-component training programs, multicomponent training programs including agility, balance, and flexibility, appear more effective in reducing ACL injury rates [12]. Other reasons for the findings could be a lack of knowledge, expertise, or resources for proper PTP. To facilitate successful PTPs, education of athletes, expert coaches, and varied resources are essential.

Limitations and future research directions

While this study offers valuable insights, it acknowledges several limitations and suggests avenues for future research. Further investigation is warranted to explore the underlying factors contributing to ACL injury awareness and incidence disparities among athletes across different sports types. Longitudinal studies evaluating the efficacy of educational interventions and injury prevention programs in reducing ACL injury rates are imperative. In addition, research into biomechanical and physiological variances among athletes in diverse sports may yield valuable insights into injury mechanisms, informing the development of targeted prevention strategies.

## Conclusions

Given the ACL injury rate among the participants, there is a pressing need for tailored educational programs targeting ACL injury prevention across various sports. These initiatives should prioritize increasing awareness of ACL injury risk factors, proper prevention training programs, and the importance of early intervention and rehabilitation. Effective implementation of such programs could significantly reduce ACL injury rates and improve overall athlete safety and performance.
